# A Computational Analysis Framework for Molecular Cell Dynamics: Case-Study of Exocytosis

**DOI:** 10.1371/journal.pone.0038699

**Published:** 2012-07-11

**Authors:** Wenhai Chen, Wen Zhou, Tian Xia, Xun Gu

**Affiliations:** 1 School of Life Sciences and Center for Evolutionary Biology, Fudan University, Shanghai, China; 2 College of Mathematics & Information Science, Wenzhou University, Zhejiang, China; 3 Department of Mathematics, Iowa State University, Ames, Iowa, United States of America; 4 Electrical and Computer Engineering Department, Iowa State University, Ames, Iowa, United States of America; 5 Biomedical Informatics Center, Northwestern University Clinical and Translational Sciences Institute, Chicago, Illinois, United States of America; 6 Department of Genetics, Development, and Cell Biology, Iowa State University, Ames, Iowa, United States of America; University of California, Davis, United States of America

## Abstract

One difficulty in conducting biologically meaningful dynamic analysis at the systems biology level is that *in vivo* system regulation is complex. Meanwhile, many kinetic rates are unknown, making global system analysis intractable in practice. In this article, we demonstrate a computational pipeline to help solve this problem, using the exocytotic process as an example. Exocytosis is an essential process in all eukaryotic cells that allows communication in cells through vesicles that contain a wide range of intracellular molecules. During this process a set of proteins called SNAREs acts as an engine in this vesicle-membrane fusion, by forming four-helical bundle complex between (membrane) target-specific and vesicle-specific SNAREs. As expected, the regulatory network for exocytosis is very complex. Based on the current understanding of the protein-protein interaction network related to exocytosis, we mathematically formulated the whole system, by the ordinary differential equations (ODE). We then applied a mathematical approach (called inverse problem) to estimating the kinetic parameters in the fundamental subsystem (without regulation) from limited *in vitro* experimental data, which fit well with the reports by the conventional assay. These estimates allowed us to conduct an efficient stability analysis under a specified parameter space for the exocytotic process with or without regulation. Finally, we discuss the potential of this approach to explain experimental observations and to make testable hypotheses for further experimentation.

## Introduction

Exocytosis is the fundamental physiological process that leads the traffic of vesicles to fuse with the plasma membrane, releasing its vesicle contents into targeted cells that control many cellular processes [1]–[3]. Substantial studies have shown that it involves multiple steps from vesicle trafficking, docking, priming to fusion [1]–[14]. During this process, a set of proteins called SNARE proteins occupy a central position in the fusion by protein-protein interacting between vesicular-specific and (membrane) target-specific SNARE protein isoforms, denoted by vSNARE and tSNARE, respectively. Moreover, this SNARE-mediated fusion is highly regulated through different modes [6], [15]–[23]. For instance, one mode is through the protein-protein interaction with MUNC18, a member of Sec1/Munc18 (SM) protein family, while the other mode is through the Ca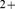-triggered exocytosis [14], [15], [5], [20].

Although experimental studies have provided invaluable insights for the underlying exocytosis mechanisms, the process of exocytosis is a typical example to show the difficulty in conducting an analysis at the systems biology level [24]–[28]. That is, while the biochemical reaction chain is straightforward and simple, the regulation *in vivo* of the system is complex. As many kinetic rates are unknown, and concentrations of proteins, complexes and substrates keep changing in both *vivo* and *vitro* environments, a biologically meaningful, global system analysis is intractable in practice. Earlier, Mezer et al. [10] proposed a computational platform to model the exocytotic process. They formulated these protein interactions into a sequential (feed-forward only without any regulation) interaction pathway to describe the exocytotic system dynamics.

In this paper, we utilize the exocytotic process as a model system to present a computational framework for system modeling and analysis. Similar to [10], we model the dynamics and architecture of the complex system by the ordinary differential equations (ODEs). First, we model the whole system by taking the regulatory elements into account. Second, we use a math techniques called inverse problem to estimate the rate parameters for the basic steps of biochemical reactions. Through the method, we are able to recover and optimize these parameters based on limited *in vitro* experimental data. Third, based on the above estimates, we can therefore approximately study the stability behavior of this system with and without MUNC18 regulation. We then attempt to explain experimental observations about different fusion efficiency caused by the change of SNARE proteins’ concentration and multiple complexes in the SNARE-induced membrane fusion. Moreover, we make a few interesting predictions that can be verified by further experimentations.

## Results and Discussion

### The Protein Interaction Network of Exocytosis

**Figure 1:**
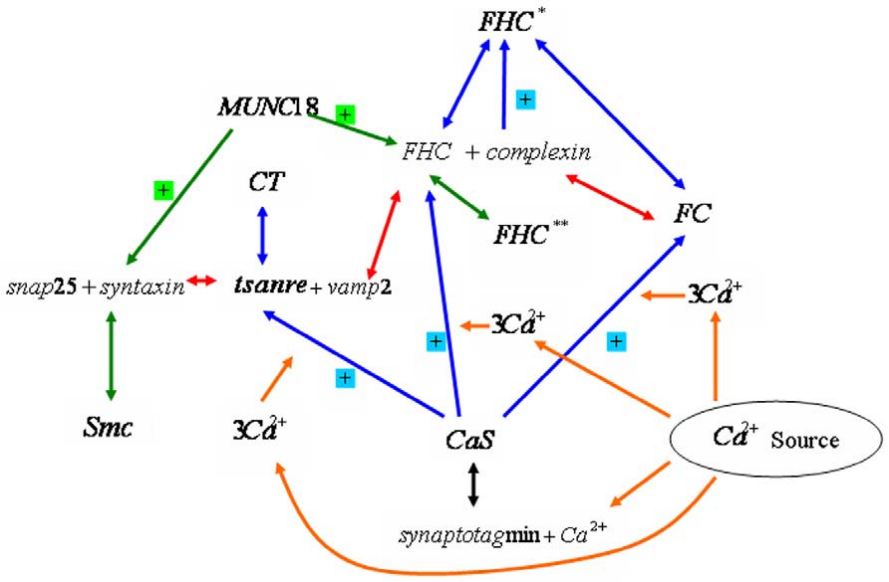
The whole process of fusion used in the mathematical model is shown. One direction arrows and symbol of 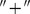 represent the reaction between proteins, ions and complexes, while full direction arrows connect two parts of a single reaction. Modified from [1]–[6].

From the view of gene network, the exocytotic process is a sophisticated combination of sequential interactions of well-defined proteins and protein complexes [1]–[13]. As shown in Fig.1, it has three major components. The first step of the basic reaction component includes two membrane proteins, SNAP25 (synaptosome-associated protein, 25 kDa) and syntaxin, together forming the so-called tSNARE; here 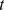 means target, the plasma membrane where the vesicle is heading for. Another important protein is vesical-associated membrane protein (VAMP2), belonging to the category of vSNARE (vesicle). In the second step, the protein complex formed by tSNARE and vSNARE is the fundamental step for the membrane fusion. In our study we consider two regulatory components, which are MUNC18-mediated and Ca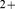-dependent regulation pathways, respectively. On the other hand, from the view of systems biology the mechanism of this exocytotic process is a dynamics system capturing the temporal change of the concentrations of proteins and intermediate complexes, which can be formulated based on an ODE dynamic system, as shown below in details.

#### The basic steps

The well-known foundations [4], [3], [8] for this exocytotic processes are the following two reactions.
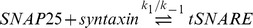


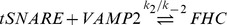
where the protein complex FHC stands for the four-helical bundle. Formation of FHC complex is the main step to promote membrane fusion, an essential part of exocytosis. In addition, there are several follow-up complex modifications. For instance, the function of complexin is as a clamp [20], resulting in



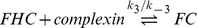
where FC is the generic notation for the protein complex of FHC and complexin.


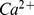
 **-dependent regulation.** 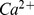 is the main trigger for the initiation of intracellular exocytosis [4], [14], [15], [5]. Suggested by [16], [4], the regulation of 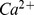 is executed through stimulating synaptotagmin. A well-known mechanism is that 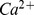 binds with the SNARE complexes (FHC) and stimulates the fusion [2]. The reaction equations to characterize the mechanism regarding 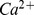 and synaptotagmin are given by
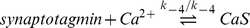



where CaS stands for the complex of synaptotagmin and one Ca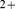 ion, and
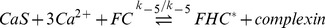


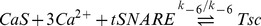


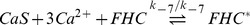



where the generic notation Tsc represents the protein complex of tSNARE and synaptotagmin binding four Ca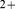 ions, and FHC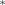 represents the complex of FHC and synaptotagmin binding four Ca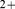 ions.

#### MUNC18-dependent regulation

MUNC18 is an important regulatory protein for the exocytotic system [6], [17], [22], through two different modes: (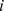) MUCNC18 associates with syntaxin to remove them from the assembly into the SNARE complexe at the beginning stage; and (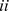) MUNC18 stimulates the fusion process by associating with FHC. These two reaction mechanisms can be written as follows.
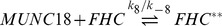
where FHC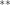 is the complex of MUNC18 and FHC to help the fusion process; and



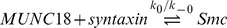
where Smc is the generic name for the protein complex of MUNC18 and syntaxin.

### Mathematical Modeling for the Whole Exocytotic System

Putting together, we have formulated a mathematical model by the ordinary differential equations (ODE) to capture how the concentrations of different proteins and complexes vary with time and how they interact each other. Based on the law of mass action and Michaelis-Menten Kinetics, and using the conventional notation 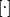 for the concentration, the ODE system is given by.
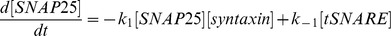


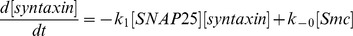


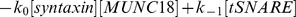


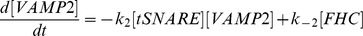


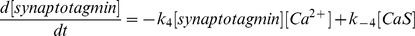


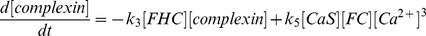


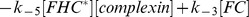


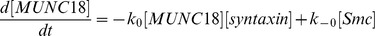





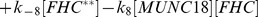


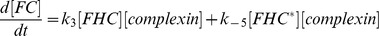


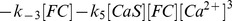


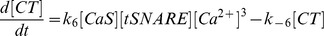


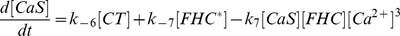


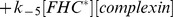


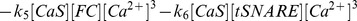


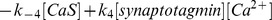


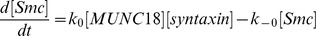


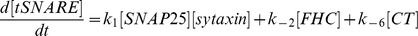


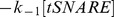


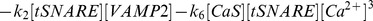


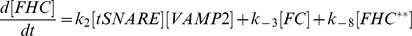


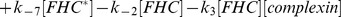


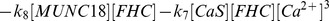


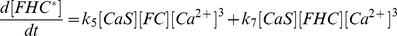


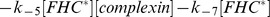


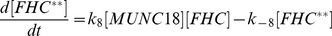
(1)


One may raise the question, due to the complexity of this network, whether we have empirical evidence enough to show the concept we try to put forward. In a recent article, we [29] have conducted a comparative network motif analysis for the Sec1/Munc18-SNARE regulatory mechanisms through a comprehensive compile of experimental data from different species and different cell types. In spite of some differences in details that have been shown important for cell-specific and species-specific system behaviors, we confidently conclude that Eq.(1) may conceptually represent the basic dynamic system that is likely universal. Some comments about Eq.(1) are presented below.

#### Dynamics of 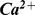


The dynamics for 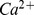 in exocytosis is complex. To be analytically feasible, we assume that during the fusion process, concentration of 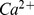 ions at active zone is temporal dependent. Thus, the dynamics of 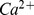 ions around the region of fusion (active zone) can be characterized as.
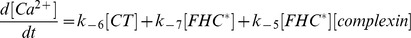


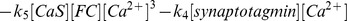


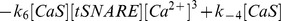


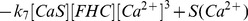
(2)where 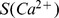 is the recruitment source of calcium. Nevertheless, the *in vivo* concentration of Ca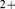 ions may stay at a roughly constant level as both external and internal sources may have kept the balance of Ca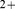. In this case, Eq.(2) can be replaced by the simplest form 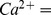
Constant.

#### Self-association of syntaxin

We notice that self-association of syntaxin is possible such that 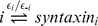, where 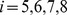, syntaxin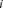 represents the complexes made of 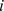 syntaxins [12]. Hence, if we take the effect of self-association into account, the system of Eq.(1) needs to be modified as follows: we have the equation for the concentration of syntaxin.
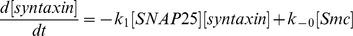


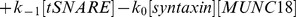


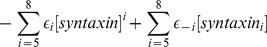
(3)and additional four equations to describe the dynamics of self-associated complexes, that is,
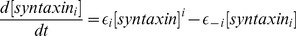
(4)where 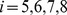


#### Mass conservation

One can show that the ODE system of Eq.(1) complies with the detailed balance principle and the mass conservation. For instance, because the only products of the reactions involving MUNC18 are Smc and FHC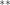, the change of concentration of MUNC18 is only relevant to the concentrations of these two complexes. Indeed, for the subsystem that only involves MUNC18, Smc and FHC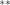, we obtain.
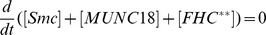


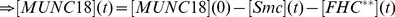
(5)since there are no Smc and FHC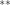 initially.

#### Spatial effect

Denote all of the variables (concentrations) in Eqs.(1)–(5) by a vector 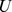 so that the ODE system can be rewritten in a concise form of 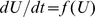, where 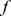 is vector of functions on the right hand side of each equation. If the spatial effects of proteins and protein complexes are considered, this system should be generally written as follows.
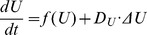
(6)where 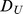 stands for the vector of diffusion coefficients of proteins, complexes and ions. Study of reaction diffusion equations described by Eq.(6) would be interesting particularly for the problems related to the developmental process.

### Estimation of Reaction Rate Parameters

The whole system for the exocytotic process as described in Eq.(1) is a typical example to show the general difficulty in systems biology [24]–[28]. While the biochemical reaction chain is simple, the regulation *in vivo* of the system can be very complex. In addition, most paremeters remain unknown in this ODE system, including the initial concentrations of different proteins and complexes, and the reaction rates in both *vivo* and *vitro* environments. Hence, it is almost intractable in practice to carry out a global system analysis. As a first step to overcome this difficulty, we attempt to estimate the rate parameters for the basic steps of exocytotic process. Among different methods, we choose the technique of inverse problem that has two advantages: the required data size is small, and the algorithm guarantees the uniqueness and efficiency [25].

#### The fundamental subsystems

As the well-known machinery, chemical reactions.
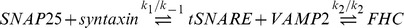
are fundamental for membrane fusion. The behavior of this subsystem involving only proteins SNAP25, syntaxin and VAMP2 can be described as



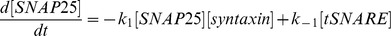


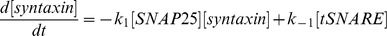


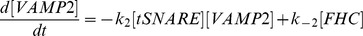


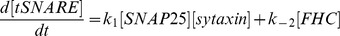


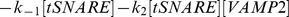


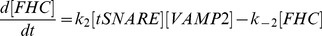
(7)


Using the inverse problem technique [30] to estimate rate parameters requires initial concentrations. In the following the symbol 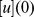 is used for the concentration of variable 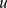 at time 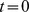. From the experimental data [9], we set the initial condition for system Eq.(10) to be: 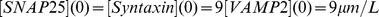, and 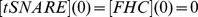. It should be noticed that the estimation of kinetic rate parameters are usually insensitive to the initial conditions, as verified by our simulation studies (not shown).

#### Reparameterization for data-fitting

In the experimentation, researches use the fluorescence intensity, 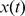 to measure the time-dependent fusion process. The relationship between the concentrations of core complexes (FHC, FHC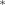, FHC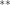) and the fluorescence intensity, 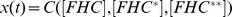, needs to be addressed in some details. Some experimental studies such as [11] suggested that the function 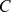 can be roughly considered to be linear when the signal strength is far below the saturated level. In this case we have.
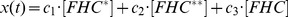
(8)where 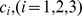 are unknown constants. We further assume that the generated intensity of fluorescence due to fusion is 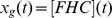 and the measured intensity of fluorescence is 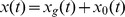, where 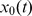 is a constant supply for fluorescence, resulting in
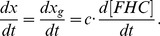
(9)Employing Eqs.(8)-(9) in the system Eq.(7), denote 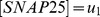, 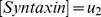, 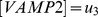, 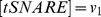, 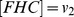 (or 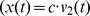), we have



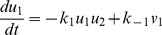


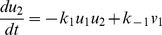


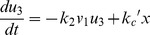


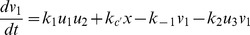


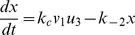
(10)where 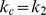 and 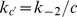. Thus, parameter recovery for the fundamental subsystem equivalent to identify the parameters 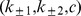 of Eq.(10).

#### Estimation by the inverse problem algorithm

**Figure 2:**
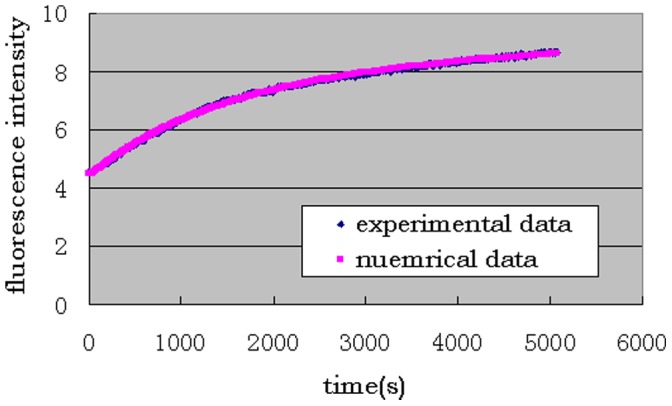
A comparison to the good-of-fit level between the numerical results by the inverse problem analysis and the original experimental data from [9]. The error is about 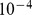.

To recover the appropriate reaction rates, we apply technique introduced by [27] to Eq.(10). Some useful theorems are presented in the section of Materials and Methods. Using the data from [9], the identified parameters are shown in the table 1. We compare the numerical results based on the identified parameters with experimental data in Fig.2, and the error is 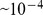.

**Table 1:** Reaction rates for the fundamental subsystem.

Reaction rates	Estimated interval (95%)	From references
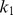	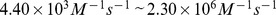	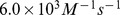 , [18]
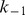	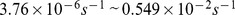	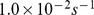 , [21]
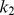	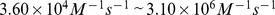	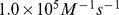 , [19]
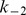	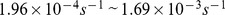	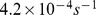 , [23]
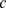	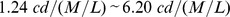	Not available

Note: 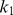 is the reaction rate for 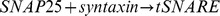; 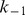 is for 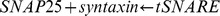; 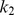 is for 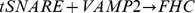, 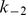 is for 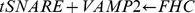, and 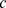 is the fusion-concentration constant.

### Stability Analysis of the Fundamental Subsystem

Estimation of rate parameters of the subsystem Eq.(10), as summarized in Table 1, allows us to carry out the stabilizing analysis under a specified parameter space. Considering the subsystem Eq.(10) with 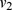 instead of 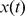, we first study the fundamental subsystem without any regulation, under the initial concentrations 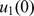, 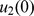, 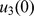, 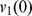 and 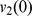 for proteins SNAP25, Syntaxin, and VAMP2, and protein complexes tSNARE and FHC, respectively. While the formal mathematical treatment is shown in the section of Data and Methods, below we discuss about the biological interpretations.

Our analysis has shown that the final steady state level of the fusion is highly dependent on initial concentrations. Obviously, three proteins (SNAP25, syntaxin and VAMP2) must exist at 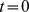 so that 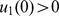, 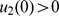 and 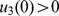. It is reasonable to assume no any fusion (here measured by FHC) at the initial time point, which means 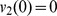. The only case we have to deal with carefully is the initial concentration of tSNARE complex, 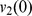. This is because in *vivo*, tSNARE is already preformed in the plasmic membrane; and then carried by vesicles, vSNARE (VAMP2 in our case) binds with it to generate fusion. In this sense, we assume 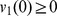 in general.

To be concise, we define 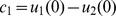, 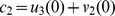, and 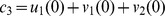 and 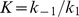. Let 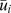 (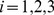) and 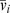 (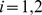) be the steady-states for 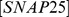, 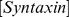, 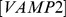, 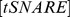 and 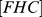, respectively, and the steadt state vector 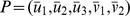. Our goal is to obtain the analytical form of 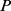. As shown in the section of Data and Methods, our mathematical analysis considers three cases under the specified parameter space given by Table 1.

1. Case-A assumes that the initial concentration of SNAP25 and syntaxin are the same such that 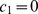. Denote 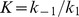, provided 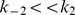, we have shown there are two locally stable-steady states, denoted by 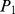 and 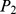, respectively, corresponding to 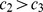 or 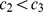. If 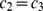, the degenerated steady state 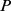 is also stable.
2. Case-B studies the problem without the assumption of same initial concentration of SNAP25 and syntaxin. Our stability analysis shows that, provided 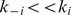 where 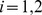, there are four steady states of 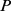 that are locally stable, corresponding to (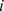) 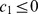 and 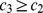, (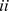) 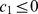 and 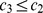, (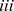) 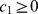 and 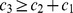, and (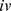) if 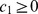, 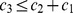, and 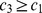, respectively.
3. Case-C considers a more general case that the reaction ratio 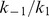 and concentrations of SNARE proteins and complexes are in the same order, that is, 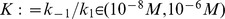 and 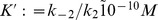. It has been shown taht the steady-states are locally stables under the following conditions: (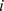) 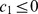 and 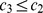; (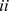) 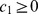, 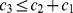 and 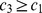; and (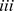) 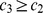 and 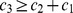, respectively.

Since we are mostly interested in the final steady state-level of fusion, i.e., 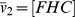, the biological meaning of above stability analyses can be summarized in Table 2. In short, for the system involving SNAP25, Syntaxin and VAMP2, we should only consider two types of initial conditions: If the initial conditions only include initial concentrations of SNAP25, Syntaxin and VAMP2, but no tSNARE, the final steady state of fusion 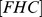 is equal to the least initial concentration of SNAP25, Syntaxin and VAMP2. In the case of non-zero initial concentration of tSNARE (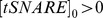), however, the final steady state 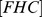 can be much higher as long as the initial concentration of VAMP2 (vSNARE) is sufficiently large. This case is particularly interested because in *vivo*, SNAP25 and Syntaxins may have been already preincubation (preformed) into tSNAREs on the plasmic membrane, before vSNARE proteins (VAMP2 in our case) approach, as carried by vesicles.

**Table 2:** A brief summary for the stabilizing analysis of the fundamental subsystems without regulation.

Initial condition for the first reaction	Initial condition for the second reaction	Fusion level at steady state, [FHC]
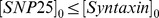	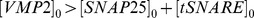	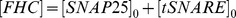
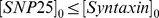	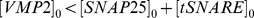	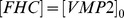
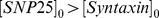	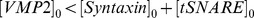	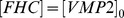
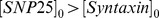	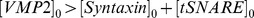	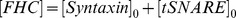

**Figure 3:**
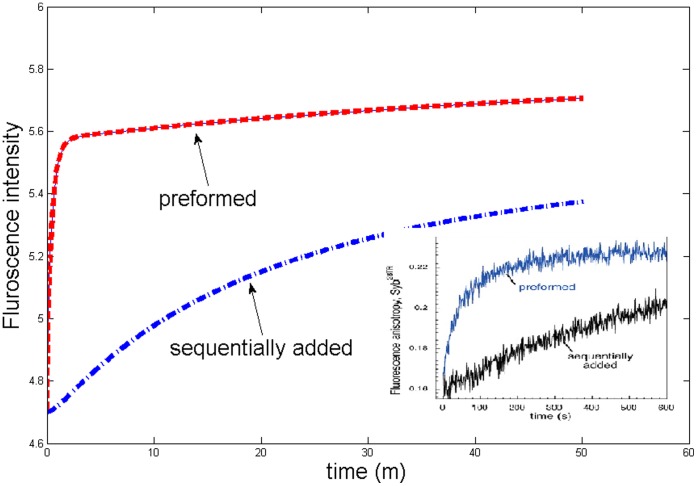
A comparison between proformed and sequential fusion processes. Numerical simulation results are presented, whereas the experimental results are in the embedded plot from [21].

Our analysis explains why the outcome of fusion process depends on the way to put these three proteins into the system [21]. One is the sequential process: SNAP25, Syntaxin and VAMP2 proteins are added into the system in order such that virtually no tSNARE protein complex has been formed when the reaction begins. The other one is the preformed process: After SNAP25 and Syntaxin proteins have been preincubation (preformed) into tSNARE, VAMP2 proteins are then added to initiate the fusion reaction. Numerical simulations have shown that the preformed process reaches the steady state much faster than the sequential one (Fig.3), which is consistent with *in vitro* experimental data (the embedded panel) [21].

### Stability Analysis on MUNC18-dependent Regulation

We furthermore study the stability behavior of the system involving the regulatory protein MUNC18. As discussed above, the MUNC18-dependent regulation has two types: (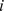) It binds tightly to a closed conformation of sytanxin that precludes the syntaxin’s involvement in the fusion process, suggesting that MUNC18 inhibits fusion by regulating the formation of tSNARE. And (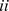) it can assemble with SNARE complexes (FHC) to accelerate membrane fusion in late stages when the concentration of four helical bundles (FHC) is high enough.

We make the following assumptions to simplify the subsystem with MUNC18-dependent regulation. Considering the situation that tSNARE has been preformed and reaction of SNAP25, sytanxin and tSNARE has reached the equilibrium, we claim that the function of MUNC18 can be characterized as follows.
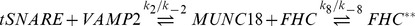
where FHC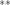 is the complex of MUNC18 and FHC, and it behaves similar to FHC to help the fusion process; and



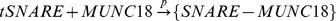
where 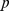 stands for the binding rate of MUNC18 onto the syntaxin in closed conformation. In the above reactions, the concentrations of four helical bundles, FHC, and four helical bundles binding with MUNC18, FHC** reflect the level of fusion. It has been shown that the disassociation rate of MUNC18-syntaxin complex is very small comparing to the binding rate, so that the second reaction is considered as an irreversible one.

Introducing variables 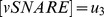, 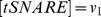, 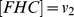, 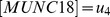, and 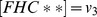, the subsystem involving MUNC18 is rewritten as.
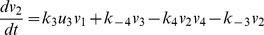


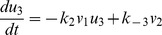


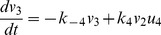


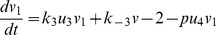


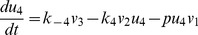
(11)


Using the mathematical approaches similar to the case of no regulation, we have studied the stability of system Eq.(11). Assume the binding rate 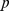, reaction rates 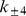 are in the range given by the reference [17]–[19], we have shown the existence of steady states of Eq.(11), including bi-stability. As the result has been rigorously presented in the section of Data and Methods, we are mainly interested in the final fusion level, as measured by 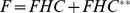. Under the assumption that the initial concentration of FHC** is zero, i.e., 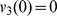, we interpret our results as follows.

(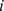) If 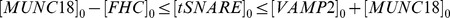, there exist two bi-stable states for the final fusion levels: One is the high fusion level, which is given by.
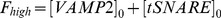
(12)


In this case, at the steady state, the concentrations of free MUNC18 and free vSNARE (VAMP2) are virtually zero, which mean all of these proteins exist in the form of FHC and/or FHC**. The second steady-state is the low fusion level (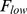), the up-bound of 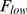 is actually 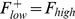, whereas the low-bound is given by.
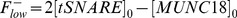
(13)


On the other hand, the low steady state fusion level, 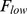 is somewhere between 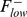 and 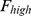. In this case, the steady state, the concentrations of free MUNC18 and free tSNARE are virtually zero, which mean all of these proteins exist in the forms of FHC and FHC**.

(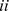) Otherwise, there exists only one steady state that is locally stable, and the final fusion 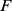 is somewhere between 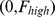.

Hence, with the regulation of MUNC18, the steady states of final level of fusion is controlled by the initial concentration of MUNC18: The behavior of bi-stability exists only when the initial concentration of MUNC18 is intermediate, whereas the boundary is determined by initial concentrations of tSNARE, VAMP2 and FHC. A lower or higher 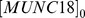 results in a single steady state of the final fusion level. Moreover, the final fusion level depends on 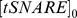, suggesting that the preincubation (preform) of tSNARE is an important factor. Indeed, using numerical simulations, we have shown that for the system involving SNARE proteins, complexes and MUNC18, preformed assays have two advantages over the sequential one: first, preincubation advances reaction rates; second, preincubation support more fusion than sequential assays. Finally, we comment that the regulation mechanism of MUNC18 may be threshold dependent, i.e. there exists an optimal threshold ? which depends on the initial concentration of tSNARE and four helical bundle only, such that MUNC18’s regulation function during fusion is maximized when the initial concentration of MUNC18 reaches the threshold (Xia et al, unpublished results).

### Conclusive Remarks

In this study, we present a framework for modeling protein interaction network which are involved exocytotic process. The framework is based on classic chemical kinetic model that generates insights into system dynamics and stability. The computational experiments and mathematical analysis reveal that the frame reconstruct biological experimental observation successfully and is able to provide useful predictions.

## Methods

### Simulation Procedures

The kinetics simulation and analysis of the whole system or the subsystems were implemented in Matlab7.0R. Differential equations were solved using the ODE23s routine. For testing the robustness of parameters, we generated 2000 random parameter sets using Latin Hypercube Sampling when all parameters are varied 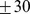
% relative to their original values, with a a uniform distribution for each parameter.

The concentrations of reactant proteins are given in molar units. For non-soluble proteins such as vSNARE and VAMP2, we followed the work in [10] and based the protein concentration estimation on the concentration of secretory vesicles in molar. During the exocytotic process, the size of vesicle pools varies with respect to different cell types from 200 to 3000. Hence, the molar concentration of vesicles was estimated in the range of 0.2–30 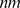. Accordingly, the concentration of VAMP2 is considered to be in an identical range of vesicle concentration (0.2–30 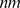) [10]. The tSNARE proteins such as SNAP25 and syntaxin are thought to be vastly expressed in vivo and the studies [1]–[6] evaluated the concentration of these protein in a range of 0.1–100 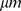. The essential regulatory protein Munc18 is known to be expressed at much lower levels, compared to SNARE proteins, with the concentrations in range of 1–30 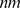 [3]–[4], [10].

### Algorithm for the Estimation of Rate Parameters

To recover the appropriate reaction rates, we apply technique of solving the inverse problem introduced by [27] to Eq.(10). Some useful results are presented below. To be concise, the ODE system Eq.(10) is written as 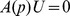, 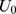 is the initial conditions, and the parameter set 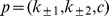.

The inverse problem claims that the parameter identification of Eq.(10) is equivalent to the optimization problem of.
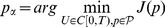
(14)subject to 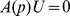 and 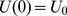, where 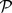 is the parameter space in 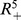 and 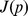 is regularized energy functional
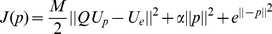
(15)where 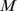 and 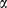 are Tikhonov regularization parameter [25], 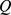 is parameter-data mapping, 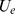 is experimental data, and 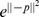 is the penalty function to guarantee the positivity of reaction rates.

The rational of the inverse problem is based on the following theorem: Suppose the solution of Eq.(10) 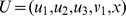 is smooth, where 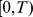 is the observation time. Then, given observed data on each time point in 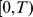, the parameters identified by the inverse problem are locally unique with respect to the initial condition. Under the assumption that all of the reaction rates are roughly constant, the optimization problem is solved through a gradient-based method. The brief algorithm is sketched below:

1. Given initial condition 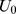, solving ODE system 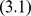 by fourth order RK and mapping it on the observation data set,
2. Gradient representation: using forward difference to approximate 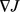,
3. Applying steepest descent to approach the global minimum starting with some initial guess,
4. Using adjoint scheme to approximate Hessian 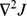 of 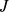,
5. Using the approximate solution given by step 2 as initial guess , and using Quasi-Newton method with 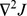 to find the appropriate parameter.

### Stabilizing Analysis of the Fundamental Subsystem

The formal claim from the stabilizing analysis of the fundamental subsystem Eq.(10) is as follows: Define 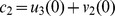, and 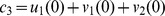 and 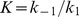. Let 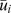 (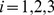) and 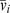 (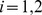) be the steady-states for 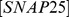, 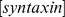, 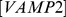, 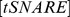, and 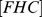, respectively, and the steady-state vector 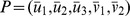.

#### Case-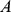


Assume the initial concentrations of SNAP25 and syntaxin are the same so that 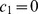. Denote 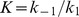, provided 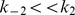. There are two stable steady states, 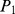 and 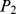: (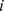) If 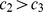, we have.
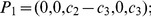
(16)and (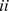) otherwise



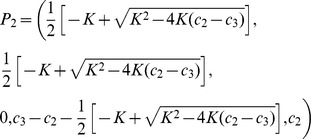



If 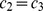, the reduced steady state is 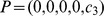, which is locally stable.

#### Case-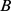


Consider the case of 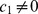. Provided 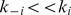 where 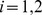, there are four steady states:

1. if 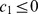 and 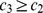, the steady state is 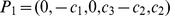 and it is stable locally;
2. if 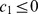 and 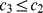, the steady state is 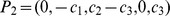 and it is a stable node locally;
3. if 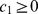 and 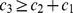, the steady state is 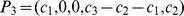 and it is stable locally;
4. if 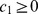, 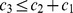, and 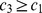, the steady state is 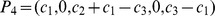 and it is a stable node locally.

#### Case-C

A more general case is that the reaction ratios (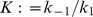, 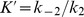) and concentrations of SNARE proteins and complexes are in the same order, i.e., 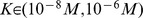 and 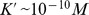. The steady states are.
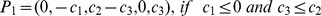


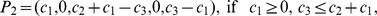


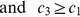
(18)and if 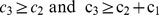


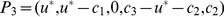
(19)where



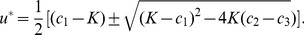
(20)Note that 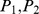 and 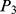 are locally stable nodes. When 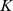 is small sufficiently comparing to the concentrations of SNARE proteins and complexes, 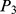 is reduced to.
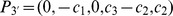


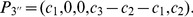
(21)


**Proof.** A concise proof is presented below. From the definition of 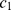 to 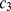, straightforward calculation simplifies the fundamental subsystem Eq.(10) as follows.
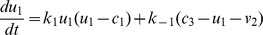


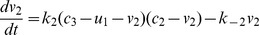
(22)


For Case-A that 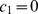, Eq.(22) can be further simplified to be.
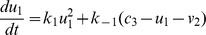


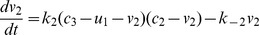
(23)


Notice that reaction rates recovered from the experimental data imply 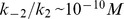, so that compared to the concentrations of SNARE complexes, 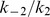 is negligible. Thus, the 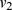-nullcline determined by Eq.(23) so that.
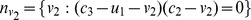



Denote 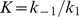, 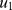-nullcline is given by.
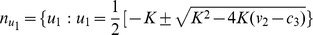



The steady states are yielded by intersecting the nullclines, and the biological interesting steady states are therefore given by 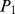 and 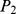. Straightforward calculation implies the steady states 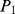 and 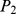 are a pair of opposite vertexes, and the relationship of 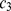 and 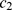 determines the choice of these two steady states. If 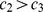, the only possible steady state is 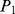; if 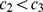, the only possible steady state is 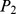.

To investigate the stability of those steady states, we calculated the corresponding Jacobian for system Eq.(23) and then evaluate the two eigenvalues, denoted by 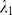 and 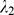, respectively. For steady state 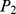, two eigenvalues for the Jacobian have no zero real part because of 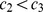, so that steady state 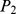 is a hyperbolic point of system Eq.(23). By Hartman-Grobman theorem, there exists a homeomorphism mapping the trajectories of Eq.(23) in an open set containing 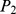 onto trajectories of its linearized system in an open set containing 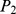. Furthermore, the homeomorphism preserves the parameterizations by time. Therefore, local behaviors of 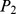 is characterized by its corresponding Jacobian, leading to 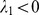 and 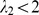. Therefore, steady state 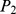 is stable locally.

For steady state 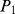, we calculated the corresponding Jacobian and showed that none of the eigenvalues has zero real part, so that 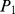 is a hyperbolic steady point of system Eq.(23). The local behavior of trajectories of (Eq.(23) in the neighborhood of 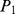 is characterized by its linearized system with respect to 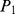. As the trace of correspondiong Jacobian is less than zero, we show 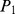 is stable locally.

In the same manner, we have shown the results presented in case-B and case-C.

### Stabilizing Analysis of MUNC-18 Regulation

For the system described in Eq.(17), there are three steady states, which are.if 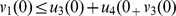 and thus 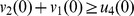,then
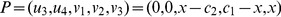
(24)for any 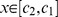, or
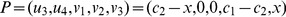
(25)for any 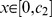.if 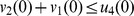, then
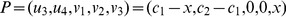
(26)for any 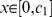.where 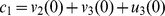 and 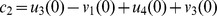. These steady states are locally stable.

*Proof:* The proof is similar to the case of system Eq.(10) without regulation.
